# Accessibility of general and specialized obstetric care providers in Germany and England: an analysis of location and neonatal outcome

**DOI:** 10.1186/s12942-017-0116-6

**Published:** 2017-12-01

**Authors:** Jan Bauer, David A. Groneberg, Werner Maier, Roxanne Manek, Frank Louwen, Dörthe Brüggmann

**Affiliations:** 1 Division of Epidemiology and Health Services Research, The Institute of Occupational, Social and Environmental Medicine, Goethe University, Theodor-Stern-Kai 7, 60596 Frankfurt/Main, Germany; 2 Institute for Health Economics and Management of Health Care, Helmholtz Centre Munich, German Science Centre for Health and Environment (GmbH), Ingolstädter Landstr. 1, 85764 Neuherberg, Germany; 3 Touro College, 500 Seventh Ave, New York, NY 10018 USA; 4 Division of Obstetrics and Fetomaternal Medicine, University Hospital of Frankfurt, Theodor-Stern-Kai, 7, 60590 Frankfurt, Germany

**Keywords:** Neonatal outcome, Obstetric care, Accessibility, Area deprivation, High-income countries

## Abstract

**Background:**

Health care accessibility is known to differ geographically. With this study we focused on analysing accessibility of general and specialized obstetric units in England and Germany with regard to urbanity, area deprivation and neonatal outcome using routine data.

**Methods:**

We used a floating catchment area method to measure obstetric care accessibility, the degree of urbanization (DEGURBA) to measure urbanity and the index of multiple deprivation to measure area deprivation.

**Results:**

Accessibility of general obstetric units was significantly higher in Germany compared to England (accessibility index of 16.2 vs. 11.6; p < 0.001), whereas accessibility of specialized obstetric units was higher in England (accessibility index for highest level of care of 0.235 vs. 0.002; p < 0.001). We further demonstrated higher obstetric accessibility for people living in less deprived areas in Germany (r = − 0.31; p < 0.001) whereas no correlation was present in England. There were also urban–rural disparities present, with higher accessibility in urban areas in both countries (r = 0.37–0.39; p < 0.001). The analysis did not show that accessibility affected neonatal outcomes. Finally, our computer generated model for obstetric care provider demand in terms of birth counts showed a very strong correlation with actual birth counts at obstetric units (r = 0.91–0.95; p < 0.001).

**Conclusion:**

In Germany the focus of obstetric care seemed to be put on general obstetric units leading to higher accessibility compared to England. Regarding specialized obstetric care the focus in Germany was put on high level units whereas in England obstetric care seems to be more balanced between the different levels of care with larger units on average leading to higher accessibility.

## Background

In the year 2000 eight millennium development goals (MDGs) were announced by the United Nations to be achieved by 2015 [1]. These goals included the reduction of the maternal mortality ratio (i.e. maternal deaths per 100,000 live births) by 75%, and universal access to reproductive health worldwide. To date, maternal mortality has been cut by almost half. However, only one in two pregnant women receive the recommended amount of care, with distinct differences in low- versus high-income countries [1, 2]. Even in developed countries, obstetric care could be at stake due to a concentration of providers towards urban areas [3]. Between 2010 and 2014, 7.2% of rural obstetric hospital units were closed in the United States [4]. In Germany, the total number of obstetric hospital units declined from 808 in 2011 to 734 in 2015 [5]. Also in England the number of obstetric care units has slightly fallen as reported in 2013 by the department of health [6]. This development may be concerning since an adequate access to obstetric care is prerequisite for satisfactory pre-, peri-, and postnatal care complying with national guidelines [7]. This access has been shown to vary geographically, not only for general obstetric services, but also for specialized obstetric care [8, 9]. There is a vast body of research assessing obstetric accessibility by catchments regarding driving times: A study conducted in the United States (US) showed that 97.3% of reproductive aged women had access to general obstetric units within a 60 min drive. This proportion decreased to 80.1% for more specialized units [8]. In England the national audit office reported a proportion of 79% of women of childbearing age living within a 30 min drive and 99.99% within a 60 min drive to both a midwifery-led unit and an obstetric unit in 2013 [9]. However, regarding specialized obstetric care, centralization has been shown to produce better survival outcomes for preterm babies requiring neonatal intensive care [10, 11]. In Germany, two levels of specialized obstetric care have been defined by the german federal joint committee: level 1 perinatal centers, which provide the most specialized care for prematurely born neonates with a birth weight less than 1250 g or less than 29 weeks gestation and level 2 units offering broader specialist care for example to newborns up to 1499 g [12]. In England three levels have been defined by the NHS: special care units (level 1), local neonatal units (level 2), and neonatal intensive care units (level 3). Neonatal intensive care units (level 3) provide the whole range of medical neonatal care for babies with a birth weight of less than 1000 g or born at less than 28 weeks gestation. On the other hand, special care units (level 1) provide only some high dependency services depending on their neonatal network and in addition they provide a stabilisation facility for babies needing a transfer to a neonatal intensive care unit. Furthermore, even though local neonatal units (level 2) provide all categories of neonatal care, the majority of babies within a local neonatal unit are over 27 weeks of gestation and do not require complex or longer-term intensive care [13, 14]. Therefore, despite some structural differences, level 1 units in Germany and level 3 units provide the most specialist neonatal care. Furthermore, level 2 in Germany and the lower levels of neonatal care in England (level 2 and level 1) provide care mainly for babies with at least 28 weeks of gestation. For the remainder of this manuscript we refer to more specialized care units as “high level” units (level 1 in Germany and level 3 in England) in contrast to low or medium level units (level 2 in Germany and level 1 and 2 in England).

Despite its common use, the term “access” often lacks an appropriate definition [15, 16]. The use of ‘access’ encompasses availability, accessibility, accommodation, affordability and acceptability of care [16]. In particular, ‘spatial accessibility’ is used to describe the combination of availability (i.e. number of obstetric care providers) and accessibility (i.e. the distance/time from demand to supply) [17]. The complexity of this concept tries to account for the many-faceted driving forces affecting a potential patient-provider contact. For instance, it allows a more profound insight into the discrepancy between the number of providers available in theory to what and how it is potentially utilized by the patients. Specifically, timely access to care is crucial for satisfactory outcomes. Hence, the spatial distribution of obstetric care providers remains an ongoing topic of discussion. This has been recognized by the German social legislation stating that in 2017 the Federal Joint Committee has to define accessibility thresholds of hospitals (defined by driving time in minutes) [18]. In Germany, the planning of health care coverage provided by hospitals is regulated on federal state level, even though adjacent states are encouraged to cooperate in order to create equal access to care [19]. Only those hospitals that are considered within this plan are refunded by social health insurances. In most states, hospital unit planning is based on the Hill-Burton formula from 1946, which considers the following area based determinants: population size, case numbers, length of stay and hospital bed utilization ratio [20]. However, modifications have been proposed to this approach [21]. So far none of these take sophisticated measures of spatial accessibility into account. In England another approach has been followed to ensure broad healthcare access: Here, neonatal care is managed via clinical networks, which were established in 2004 [22]. These networks are sought to link obstetric health care providers in order to ensure high-quality health services. Furthermore, planning of hospital beds in England is now managed by 44 sustainability and transformation partnerships (STP) [23]. Each partnership (NHS and a local council) has developed a proposal to balance supply and demand of the health care in its area. However, these proposals differ in their methods, for example using either activity or beds as the unit of planning. Therefore, a more individual and localized planning method has been applied to England compared to Germany.

With this study, we aimed to provide a robust assessment of the current state of demand and supply of obstetric care in Germany and England using a novel geospatial approach. Our objective was to (1) analyze geographical variations regarding accessibility of general and specialized obstetric units in England and Germany and (2) to examine its relationship with urbanity, area deprivation and neonatal outcome.

## Methods

Accessibility was analyzed for general and specialized obstetric units. We measured accessibility with the integrated floating catchment area (iFCA) method, which generally speaking is the ratio of the summed capacity of obstetric providers within a pre-specified distance of population location *x* divided by the summed demand for obstetric care from those providers across all locations [24]. This method is based on the two step floating catchment area (2SFCA) method [25]. However, despite its improvements compared to earlier measurement, the 2SFCA method has three shortcomings: (1) fixed catchment sizes, (2) omission of distance decay and (3) omission of competition [26]. The 2SFCA method has been modified several times to address these shortcomings. Regarding the distance decay, both stepwise and continuous approaches have been applied within the (E)2SFCA method or the kernel density function (KD)2SFCA method [26, 27]. Regarding catchment sizes, variable catchment sizes rather than fixed catchment sizes have been used within the variable (V)2SFCA method or the enhanced variable (EV)2SFCA method [28, 29]. Also, competition within the demand–supply system of healthcare has been integrated by including an additional variable by accounting for the number of competitors within a catchment [30].

The iFCA addresses limitations of earlier accessibility measurements and integrates several improvements introduced by the above mentioned methods. The iFCA integrated variable catchment sizes (i.e. the maximum distance that patients are willing to drive varies between areas), distance decay (i.e. the probability to visit a specific obstetric provider decreases with increasing distance) and competition parameters (i.e. the allocation of demand among all available GPs). The formula to be used can be displayed as follows:

1 $$AI_{x} = \mathop \sum \limits_{{y \in \left({d_{xy} \le C_{x}} \right) }} \frac{{S_{y} \cdot f_{adj} \left({d_{xy}} \right) \cdot f_{con} \left({d_{xy}} \right)}}{{\mathop \sum \nolimits_{{x \in \left({d_{xy} \le C_{x}} \right)}} B_{x} \cdot f_{adj} (d_{xy}) \cdot Huff_{x}}}$$

*AI*_*x*_ is the accessibility index at birth location *x*. Therefore *AI*_*x*_ represents accessibility from the population point of view. Birth locations were defined as cell centroids of a km^2^-population grid [31]. *S*_*y*_ represents the capacity (live birth volume) of obstetric care providers at location *y* (i.e. the care provider characterized by its location). For general obstetric units the capacity was defined as the birth volume per year and for specialized units as the number of episodes per year*. B*_*x*_ is the birth volume per year at birth location *x*. Distances *d*_*xy*_ (in minutes by car on public roads) between birth locations and obstetric providers were calculated for a maximum catchment size of 120 min (*C*_*max*_). In the primary care sector a maximum catchment size of 60 min has often been used [32]. Furthermore, the same catchment size has been used for the obstetric sector [8, 9]. However, it has been shown that especially in rural areas the maximum travel time to a general practitioner can be up to 120 min [33]. Since obstetric care is spatially more scares compared to general practitioners we applied a maximum catchment size of 120 min to obstetric care in order to include all relevant care providers. *f*_*adj*_(*d*_*xy*_) is the adjusted and *f*_*con*_(*d*_*xy*_) the constant distance decay function. Both functions represent a downward sigmoid function. *f*_*adj*_(*d*_*xy*_) is adjusted to the distance distribution (median and standard deviation; SD) of the nearest 10 general units (specialized units: nearest five) for each birth location *x*.

2 $$f_{adj} \left( {d_{xy} } \right) = \frac{{1 + e^{{ - \frac{{\left( {Median} \right) \cdot \pi }}{SD \cdot \sqrt 3 }}} }}{{1 + e^{{\frac{{\left( {d_{xy} - Median} \right) \cdot \pi }}{SD \cdot \sqrt 3 }}} }}$$

3 $$f_{con} \left( {d_{xy} } \right) = \frac{{1 + e^{{ - \frac{{\left( {C_{max} /2} \right)*\pi }}{{SD_{max} *\sqrt 3 }}}} }}{{1 + e^{{\frac{{\left( {d_{xy} - C_{max} /2} \right)*\pi }}{{SD_{max} *\sqrt 3 }}}} }}$$

Both functions return weight values ranging between 0 and 1. With the adjusted function differing travel behaviour of patients is accounted for: For example, in rural areas with less provider availability, patients are more likely to travel longer distances compared to urban areas with no need to travel long distances. With the constant function all adjusted functions are smoothly fitted within the predefined maximum catchment. *C*_*x*_ is the effective catchment size and is defined as the distance *d* for which f_adj_(d)·f_con_(d) = 0.01. Beyond this cut off, values have been shown to be negligible within sigmoid functions approaching zero [34]. *Huff*_*x*_ reflects the probability of demand according to the Huff Model [35]. The Huff Model accounts for alternative competing obstetric services across all locations *z*, as long as those are within the effective catchment size C_x_ of birth location *x*.

4 $$Huff_{x} = \frac{{S_{y} \cdot f_{adj} \left( {d_{xy} } \right)}}{{\mathop \sum \nolimits_{{z \in \left( {d_{xz} \le C_{x} } \right)}} S_{z} \cdot f_{adj} (d_{xz} )}}$$

For the analysis of neonatal outcome and accessibility we used a facility-based approach to analyze the relationship between neonatal outcome and accessibility. The facility-based approach was applied to specialized obstetric units using the average distance from selected birth location to the unit. Birth locations were selected if there was a distance based probability (*f*_*adj*_(*d*_*xy*_) and *f*_*con*_(*d*_*xy*_)) of more than 90% to choose the respective specialized care unit. The computed average distance was the proxy indicator of accessibility. Due to data limitation this analysis is limited to specialized obstetric care units.

### Data sources

#### General obstetric units

We used data as of 2015 (location and number of live births per year) of obstetric hospital units in Germany (n = 734) and England (n = 223) including midwife-led units on the same site as obstetric units [5, 36].

#### Live birth volume per km^2^

We used the GEOSTAT 2011 population km^2^-grid provided by EUROSTAT for the allocation of birth volume in Germany and England [31]. Geocoded live birth volume was retrieved on municipality level for Germany (n = 737,575 births) and on local authority level for England (n = 664,399 births) as of 2015 [37, 38].

#### Specialized obstetric unit

We retrieved data from perinatal center level 1 (high level) and level 2 (low level) units in Germany (total of n = 221 units) and from neonatal units level 1 (low level), level 2 (medium level) and level 3 (high level) units in England (total of n = 159 units) [14, 39]. For each unit we obtained the number of neonatal episodes. In addition, neonatal outcome data were retrieved. For Germany neonatal outcome was defined by the perinatal survival (total and without severe complications). For England, neonatal outcome was defined by the number of babies with impairment after 2 years and the number of babies who were not screened on time after discharge for retinopathy of prematurity. For Germany these data represent averaged data from 2010 to 2015. For England these data were as of 2015.

#### Road network

Road network data of Germany and England were obtained from TomTom Multinet data (TomTom N.V., Amsterdam, Netherlands) as of 2015.

#### Degree of urbanization

We used the degree of urbanization (DEGURBA) defined by EUROSTAT as of 2015 to measure urban–rural differences in England and Germany [40]. The methodology of DEGURBA is identical for England and Germany and therefore has two main advantages compared to other urban–rural classifications: greater comparability and a harmonization of spatial concepts.

#### Area deprivation

As area measures for material and social deprivation, we used the indices of multiple deprivation for Germany (GIMD; as of 2010) and England (IMD; as of 2015) [41, 42]. Both indices of multiple deprivation are an overall measure of deprivation constructed by combining seven weighted domains (e.g. income, employment or education). Furthermore the overall indices represent deciles (i.e. ranking areas by deprivation and grouping in 10 equal groups) and therefore have a range of 1–10 (i.e. from the most deprived to the least deprived areas).

### Statistics

All spatial calculations and data preparations were done using ArcMap 10.4 and ArcGIS Pro 1.3 (ESRI Inc., Redlands, USA). Further statistical calculations were performed with SPSS Version 23 (IBM, Armonk, USA). We used non-parametric testing: Kruskal–Wallis-test to test for significant differences and Spearman Rho r (two tailed) for a correlation analysis. The z-score (standard score) was calculated for accessibility values. In addition, calculation of catchment size was performed with RStudio (R Core Team, Vienna, Austria) including the packages ‘rootSolve’ and ‘plyr’. Furthermore, based on the Getis-Ord Gi* statistic a hot spot analysis with false discovery rate correction was performed. The hot spot analysis was used to identify significant clusters of high and low accessibility. For the hot spot analysis we determined the scale of analysis by using the spatial distribution of birth locations based on the average distance to the 30 nearest neighbors: accordingly, a fixed distance band of 3691 m was used.

## Results

In 2015 there were a total of n = 737,575 births in Germany and n = 664,399 births in England. The annual birth volume of obstetric units was n = 722,621 in Germany and n = 649,267 in England. Therefore, 98.0% of all babies born alive in Germany (97.7% in England) were born in an obstetric unit.

The analysis of n = 213,903 birth locations in Germany and n = 89,547 in England revealed that all but n = 4 birth locations in Germany (n = 2 in England) could reach a general obstetric unit within 120 min. Even within 60 min all but n = 21 birth locations in Germany (n = 16 in England) could reach at least one general obstetric unit. Regarding specialized obstetric units, the vast majority of birth locations in England were able to reach a unit within a 120 min car ride (low level: 98.96%; medium level: 99.51%; high level 3 99.98%). In Germany, numbers were similar with 99.99% (high level) and 99.95% (low level). The descriptive results of the accessibility analysis are shown in Table 1.

**Table 1:** Descriptive results of obstetric accessibility in Germany and England

Level of care	General obstetric units		Specialized obstetric units				
	Germany	England	Germany		England		
	–	–	High	Low	High	Medium	Low
Number of facilities (n)	734	223	162	59	44	37	78
Total birth count (n)	722,621	649,267	7782	598	45,065	12,504	44,375
Mean distance (min) to closest facility [SD]	14.2 [6.9]	15.6 [7.9]	26.7 [13.3]	41.8 [21.3]	35.7 [19.8]	35.2 [19.1]	30.6 [22.6]
Mean distance (min) to closest set^a^ of facilities [SD]	32.0 [10.3]	38.5 [14.7]	45.2 [16.1]	69.6 [22.8]	60.7 [29.7]	63.2 [25.0]	50.6 [26.8]
Origin–destination Pairs (n)	25,945,405	6,966,009	6,016,565	1,934,309	1,453,655	1,029,386	2,632,833
Mean catchment area (min) [SD]	56.0 [5.6]	59.6 [8.8]	64.1 [9.4]	75.6 [9.9]	83.8 [16.7]	86.2 [14.8]	77.5 [16.2]
Mean accessibility index [SD]	16.2 [11.0]	11.6 [5.0]	0.05 [0.04]	0.002 [0.002]	0.235 [0.120]	0.069 [0.052]	0.338 [0.227]

^a^n = 10 for general obstetric units and n = 5 for specialized obstetric units

### Accessibility of general obstetric units

**Fig. 1:**
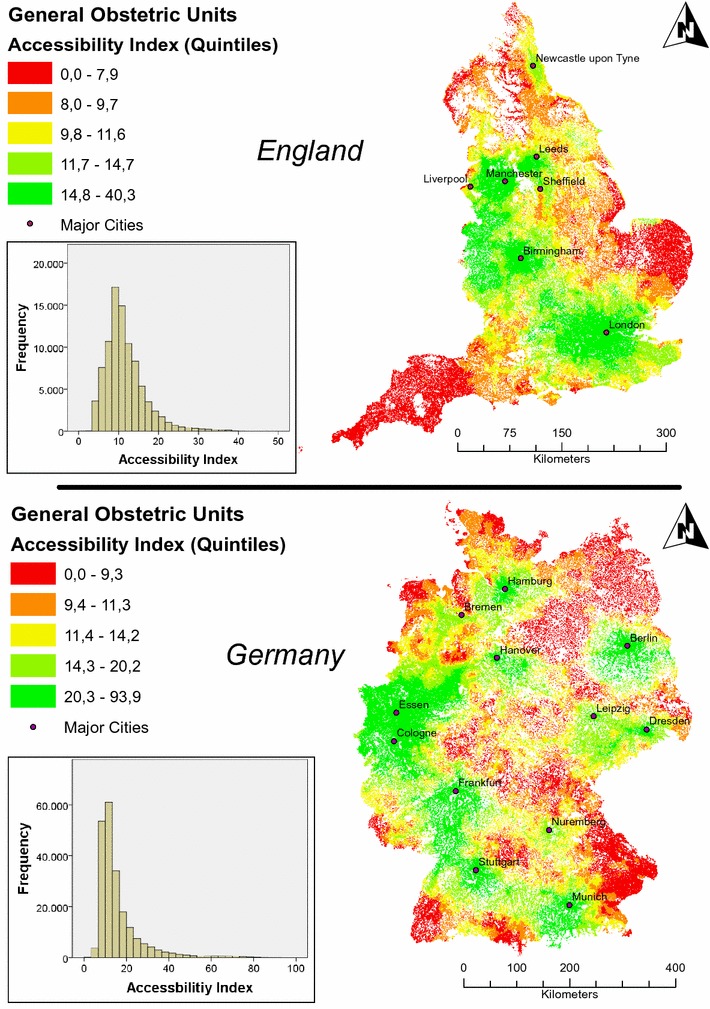
Accessibility of general obstetric units in England and Germany [Geodata source: derivative work from the authors based on data © GeoBasis-DE/BKG 2016 and data © Crown copyright and database right 2011–2015]

Accessibility in Germany was significantly higher than in England (accessibility index of 16.2 vs. 11.6; p < 0.001). Accordingly, the mean distance in minutes to the closest 10 general obstetric units was significantly shorter in Germany than in England (32.0 vs. 38.5 min; p < 0.001). This was mainly due to the relatively high number of units in Germany compared to England in relation to the birth count: On average, there were 984 births per obstetric unit in Germany and 2912 births per unit in England. Mapping of general obstetric unit accessibility (Fig. 1) revealed three main areas in England with high accessibility values: (1) London area, (2) West Midlands and (3) Manchester area. We further performed a hot spot analysis to test the visual distribution of accessibility across England for statistical significance taking neighboring birth location into account: The above mentioned three main areas had significant higher accessibility indices compared to the rest of England. Furthermore, the highest indices were present in the London area (maximum z-score: 34.6; p < 0.001).

In Germany accessibility was more dispersed. However, it has to be noted that accessibility values were less skewed in England whereas in Germany there was a skewness of distribution due to outlier towards high accessibility indices. These outliers can be explained by the partially high concentration of providers in some urban areas in Germany: for example, all birth locations with accessibility indices > 60 were located in the Cologne/Essen area (Rhine-Ruhr metropolitan region) as shown in Fig. 1. This finding was supported by the hot spot analysis with significant higher accessibility indices in the Rhine-Ruhr metropolitan region compared to other areas in Germany (maximum z-score: 39.7; p < 0.001).

Furthermore, the birth volume at each general obstetric unit was modeled based on the live birth volume per km^2^ within the accessibility analysis. The average modeled birth volume per unit in Germany was 571 (SD: 496) compared to the actual average birth volume per unit of 984 (SD: 681). This modeled birth volume was highly correlated with the actual birth volume with r = 0.91 (p < 0.001). In England an average of 1828 live births per general obstetric unit was modeled compared to the actual number of 2912 (SD: 2177). Again, the modelled birth volume was highly correlated with the birth volume with r = 0.95 (p < 0.001). Both examples indicate the utility of this approach in health care planning of general obstetric units on an international level.

### Accessibility of specialized obstetric units

In contrast to accessibility of general obstetric units as descried above, accessibility of specialized obstetric units was significantly higher in England compared to Germany on all analyzed levels of care: The mean accessibility index of units with high level of care was 0.235 in England compared to 0.002 in Germany (p < 0.001). However, for these units the mean travel distance was longer in England compared to Germany (60.7 vs. 45.2 min; p < 0.001). Therefore, despite providing shorter travel distances, accessibility was lower in Germany. Also, unit capacities in Germany were significantly smaller compared to England with a total of 101,904 live births in specialized obstetric units in England vs. 8380 in Germany (i.e. a birth volume per unit in regard to the total birth count of 15.7% in England vs. 1.2% in Germany). Furthermore, the raw unit count was also lower in Germany compared to England: 73.3% of specialized obstetric units in Germany provided the highest level of care compared to 27.7% in England. Even if medium and high level units in England were combined, their share with 50.9% of all units in England was still smaller compared to high level units in Germany.

**Fig. 2:**
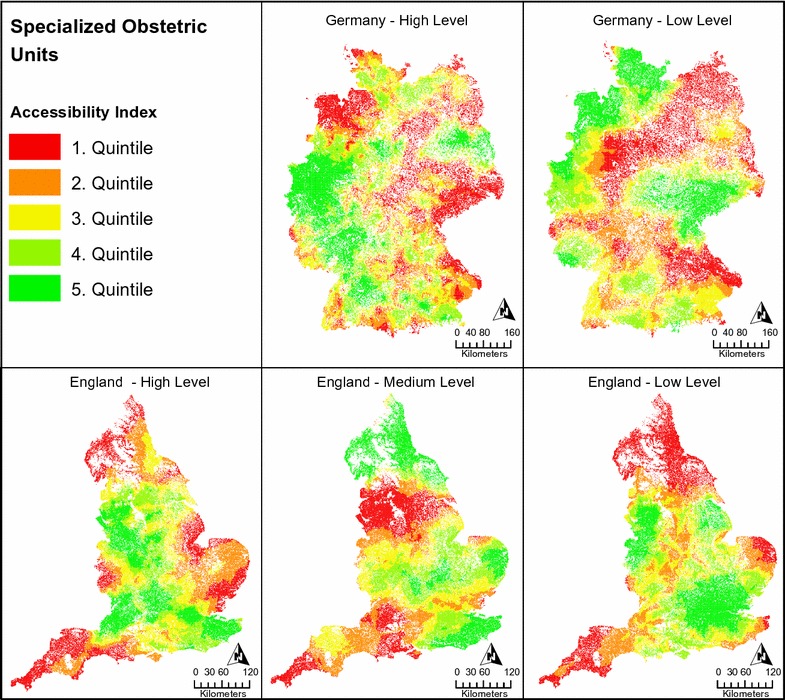
Accessibility of specialized obstetric units in England and Germany in respect to the level of care

Regarding the distribution of specialized obstetric unit accessibility, Fig. 2 shows distinct geographical disparities across the different levels of care. For high level units in Germany accessibility was more dispersed compared to low level units with several smaller high accessibility clusters especially in urban areas. Low level units on the other hand showed wide-spread high accessibility clusters especially in the North West and mid-east of Germany. In England there were high accessibility clusters for low level units especially around London and the West Midlands, for medium level units in the Midlands, East Anglia, and the north of England, and for high level units in the South East, the London area, South West and North West. In summary, depending on the level of care there were geographical disparities on national level for accessibility of specialized obstetric care in England and Germany.

### Accessibility, urbanity, area deprivation and neonatal outcome

As presented above, especially in urban areas higher accessibility indices were present for both England and Germany. This observation was supported by the correlation analysis (Table 2).

**Table 2:** Correlation analysis of obstetric accessibility in England and Germany

	Area deprivation		Urbanity		Neonatal outcome^a^	
	*r*	*p* value	*r*	*p* value	*r*	*p* value
Germany						
General obstetric unit	− 0.31	< 0.001	0.37	< 0.001	–	–
Specialized obstetric unit						
High level	− 0.38	< 0.001	− 0.32	< 0.001	0.15	0.063
Low level	− 0.03	0.003	− 0.02	0.003	0.06	0.676
England						
General obstetric unit	− 0.06	< 0.001	0.39	< 0.001	–	–
Specialized obstetric unit						
High level	− 0.11	< 0.001	− 0.33	< 0.001	0.17	0.267
Medium level	0.10	< 0.001	0.05	< 0.001	0.20	0.225
Low level	0.07	< 0.001	− 0.23	< 0.001	0.16	0.167

r = spearmen’s rho with two tailed significance

^a^For the analysis of neonatal outcome the facility-based approach was applied to specialized obstetric units

General obstetric unit accessibility was significantly correlated with urbanity with r = 0.37 (p < 0.001) in Germany and r = 0.39 (p < 0.001) in England. Therefore, accessibility was significantly higher in urban areas both in England and Germany to a similar degree. To a lesser degree, a correlation with urbanity was also revealed for specialized obstetric units with high level of care for both Germany and England and for low level units in England. However, there was no relevant correlation for low level units in Germany and medium level units in England. Looking at area deprivation, accessibility of general obstetric units and specialized obstetric units with high level of care was significantly higher in less deprived areas in Germany. In England there was no relevant correlation present regarding area deprivation. Therefore, people in Germany living in less deprived areas are possibly provided with higher accessibility of obstetric care whereas in England accessibility of obstetric care was not related to area deprivation. Regarding neonatal outcome, we found no relevant correlation with accessibility of specialized obstetric units regardless of the level of care in England as well as in Germany. The detailed results are displayed in Table 2. Even though positive correlations were present (i.e. higher accessibility and better outcomes), there was no statistical significance. Therefore, accessibility of specialized obstetric units had no statistical effect on the analyzed neonatal outcomes.

## Discussion

This high-resolution analysis demonstrated significant geographical variations of obstetric care accessibility in Germany and England for all analyzed levels of care. Accessibility of general obstetric units was significantly higher in Germany compared to England, whereas accessibility of specialized obstetric units was higher in England. We further demonstrated higher obstetric care accessibility for people living in less deprived areas in Germany. Also urban–rural disparities with higher accessibility in urban areas were confirmed for both Germany and England. In regard to neonatal outcomes, this analysis did not support an effect of accessibility on neonatal outcomes. Finally, we were able to precisely model obstetric care demand on care providers on an international level.

The presented results could support health care planning by providing insights regarding the structure of obstetric care. Furthermore, this approach could be used to forecast obstetric care demand and therefore facilitate allocation processes. By using the iFCA method to measure obstetric accessibility, we were able to apply the same methodology on an international level without having to adapt parameters manually due to differing spatial characteristics. For example, the iFCA methodology adapted to the varying willingness to travel long distances in rural and urban areas. Therefore, catchment sizes were individually computed depending on the availability of providers (i.e. catchment sizes in urban areas are smaller than in rural areas). Earlier methodologies like the 2SFCA, the E2SFCA, V2SFCA, or the 3SFCA method required a predefinition of catchment sizes or use the same decay function for the whole study area [26–30]. However, the complexity of the analysis is of concern since it limits the broad applicability of this approach. Furthermore, this conceptualization of access only addresses potential access, which is different from patients actually visiting a certain obstetric care facility [17]. Despite these limitations, this approach is based on a valid and well established model for measuring accessibility [24]. Regarding the analysis of neonatal outcomes, it has to be noted that we used a facility-based approach (outcome data on facility level), whereas other studies used a population-based approach [43, 44]. We also want to underscore that no threshold values of obstetric care accessibility have been established thus far aiming to differentiate poor access from good access. Another limitation is the omission of births delivered by freestanding midwifery units and home births. However, since the majority of births were delivered in obstetric hospital units, this omission will most likely not affect the outcome: For example only two per cent of births in England were delivered at home in 2012 [9]. In this regard, it has to be noted that in England there is a distinction between obstetric units, alongside midwifery units (units on the same site as an obstetric unit) and freestanding midwifery units [6]. Since this system is not directly transferable to Germany we classified alongside midwifery units as obstetric units due to their attachment to obstetric units, which more accurately reflects the German system. Even though this simplification of maternity services in England leads to inaccuracies regarding the national analysis in England, the improvement regarding the international analysis is substantial.

In our study 99.99% of births in Germany as well as England occurred in areas with access to at least one general obstetric unit within 60 min. In this respect, we documented accurate data in line with current literature [8, 9]. Furthermore, a better access to obstetric care in Germany and England compared to the US was shown. However, it has to be noted that the US in contrast to Germany and England is less densely populated, which effects mean travel times. Still, Marlow et al. [11] found that only 56% of extremely preterm babies in England were born in hospitals providing the highest level of perinatal care. This low rate is discussed not necessarily being due to distance: in Finland the provision of the highest level neonatal care is concentrated to five obstetric units, and still 78–95% of infants less than 32 weeks or more than 1500 g are being delivered in one of these units [45]. Similarly, in the Lazio region in Italy 89% of infants with less than 32 weeks were admitted to a level 3 unit (high level of care) [46]. Therefore other factors besides geography are likely to affect the birth location of such high risk births (e.g. maternal choice, timing of birth or insufficient referring systems).

However, geographical variations of obstetric care accessibility, as reported in our study, are in line with the current literature: The distribution of maternity units as well as higher levels of obstetric care units have been shown to vary throughout Europe with differing access due to different travel distances to the nearest provider [47]. However, conceptualizing access by simply relying on the travel distance to the closest provider falls short in several aspects, especially since this measure is insensitive for congestive areas with more than one provider available [17]. Based on modeling spatial accessibility, a study was conducted in Shenzhen (China) focusing on maternity units. Despite the differing scale (city level vs. national level as used in our study), the authors also reported significant geographical variations of maternity unit accessibility [48]. Such variations are common due to a general trend to close down obstetric departments in rural areas. In the US, this development resulted in an additional driving distance of 29 miles to reach the nearest hospital providing obstetric care in remote locations [4]. An increase of travel time due to the decreasing number of available maternity units has also been reported for France [49]. Closing down obstetric facilities will likely limit a woman’s choice of pre-, peri- and postnatal care, which may affect compliance and ultimately the course of pregnancies: It has been shown that one-third of women select their maternity unit based on proximity [50]. This proportion increased to 85% if the closest and second closest facility were more than 30 km apart [50]. Another study found that 82% of women delivering their baby in a particular hospital unit lived less than 40 miles away from this location [51]. Therefore, selecting a facility is strongly linked to its geographical availability. In our study, we both documented areas providing limited and abundant access to obstetric care facilities for pregnant women. If such an empowerment of women—to deliver their baby in facilities of their choice—complies with national health care policies, our results can be used to identify areas of limited choice. In this regard access to obstetric care in Germany and England could vary not only because of geographical differences, but because of health care system differences: In England there is the National Health Service (NHS), whereas in Germany there is mainly the Social Health Insurance (SHI). Both are mainly financed by pooled public funds via either taxes (NHS) or social insurance contributions (SHI) [52]. The system itself has a major impact on the performance of the health care system. Furthermore, as shown for the accessibility of the primary care sector, SHI systems have lower accessibility of primary care than NHS systems [53]. Therefore, the health care system in place is likely to influence accessibility of obstetric care. In our study specialized obstetric care in England was more balanced between the different levels of care compared to Germany. This may be due to differing national strategies on hospital planning as reported in the background.

The relationship between health outcome and travel time to care providers has been mainly studied in cardiologic and neurovascular emergency settings. Here, a negative impact of longer travel distances on patient outcomes has been shown [54]. However, this correlation can be extended to obstetric care in high-income countries as revealed by recent studies [43, 44, 55]. For example, Ravelli et al. [44] associated a travel distance of more than 20 min with adverse perinatal outcomes (Odds ratio: 1.27; 95% CI 1.17–1.38). On the other hand a French study found that distance to an obstetric unit did not increase neonatal mortality risk except for distances of more than 45 km [56]. Our study did not support an association of accessibility and neonatal outcome on national level using routine data in England or Germany. However, the data used for this analysis did not reflect emergency settings and represent aggregated data rather than individual data. As pointed out by Lorch et al. [7] closing down obstetric units is initially associated with adverse perinatal outcomes, which ameliorate in the long term. However, there is also evidence from Finland that there is no need to close down small hospitals in a regionalized system provided that a functioning referral system is in place [57]. In this context, it has to be stated that neonatal outcomes are modified by numerous aspects such as preexisting conditions, obesity/overweight, smoking, and older maternal age [58]. Furthermore, substandard care is influential since it has been shown to contribute to 20–30% of stillbirths in high-income countries [59]. Socioeconomic factors have further effects on the neonatal outcome—even in high-income countries such as Germany or England with universal access to essential health care: here, a lower socioeconomic status has been shown to negatively impact the neonatal outcome [60]. In our analysis, we demonstrated lower accessibility for people living in socially deprived areas in Germany, however not in England. Therefore, in Germany health inequities associated with accessibility variations seem to be present.

Our analysis focused on high-income countries. However, limited access to obstetric care is especially present in low- and middle-income countries. In 2008, more than 50% of all maternal deaths worldwide occurred in only six countries [61]. Furthermore, the maternal mortality ratio per 100,000 live births varied significantly among low- and high income countries: In 2011, the maternal mortality ratio was 21.7 in developed countries versus 335.8 in developing countries. In Germany, it was even lower than the aforementioned average with 10.8 maternal deaths per 100,000 live births [2]. Also, in regard to child mortality (deaths per 1000 live births within first 6 days) significant differences were present: In Germany, 1.4 deaths per 1000 live births were documented in 2011 (United Kingdom: 2.1), whereas 35.7 were reported in Equatorial Guinea (developed vs developing: 2.6 vs. 17.7) [2]. These data show that from a global perspective the quality of obstetric care in England and Germany is relatively high. However, the iFCA method used in the presented study has not been yet applied in low-income countries. Due to its adapting nature, the iFCA should be applicable in both high and low-income countries, but further studies are needed to test its applicability. In this context it has to be noted that the measurement of health care quality remains a delicate matter. In Germany, it is discussed to prioritize quality indicators in the process of hospital planning [62]. For general obstetric units in particular, there are already quality criteria’s in place that need to be fulfilled in order to be considered within the federal state hospital plan: For example, in four out of 16 states in Germany the minimum birth volume per year per obstetric unit is set to 300 [12]. Further seven states relate to quality criteria defined by the Federal Joint Committee such as criteria regarding employee qualifications or infrastructure [12]. In order to provide a standard of care according to national guidelines and to avoid unfavorable outcomes, e.g. the 20–30% of stillbirths linked to substandard care in high-income countries as cited above, quality criteria seem to be an appropriate approach.

The revealed differences regarding the accessibility of general obstetric units in England and Germany could be due to several factors. First, smaller unit capacities in Germany compared to England with a total of 101,904 live births in specialized obstetric units in England vs. 8380 in Germany (i.e. a quota of 15.7% in England vs. 1.2% in Germany in regard to the total birth count). Second, the raw unit count suggested that in Germany the focus of specialized obstetric care is put on facilities providing the highest level of care: 73.3% of units in Germany provided the highest level of care compared to 27.7% in England. Furthermore, there is a relatively high number of general obstetric units in Germany compared to England in relation to the birth count: On average, there were 984 births per general obstetric unit in Germany and 2912 births per unit in England. Therefore, this comparison reveals that in Germany the focus seems to be put quantitatively on general obstetric units and within specialized obstetric units on high level units whereas in England obstetric care seems to be more balanced between the different levels of care with larger units on average. As reported above centralization processes could have positive effects for health care systems in high-income countries—especially on the long term after negative effects have ameliorated. If such centralization processes are taken into consideration by health care planners, as for example proposed by the National Clinical Advisory Team in England, our study may help to facilitate such processes [6].

## Conclusion

Accessibility differences regarding obstetric care in Germany and England have not been assessed so far. With this study an in-depth approach to measure obstetric accessibility has been applied showing both significant international and national differences regarding general and specialized obstetric care in England and Germany. In Germany the focus of obstetric care seemed to be put on general obstetric units leading to higher accessibility compared to England. Regarding specialized obstetric care the focus in Germany was put on high level units whereas in England obstetric care seems to be more balanced between the different levels of care with larger units on average leading to higher accessibility. Furthermore, neonatal outcome was not related to accessibility in contrast to area deprivation.
